# Latent Variables Analysis in Person-Oriented Research

**DOI:** 10.17505/jpor.2023.25258

**Published:** 2023-06-17

**Authors:** Alexander von Eye, Wolfgang Wiedermann, Stefan von Weber

**Affiliations:** 1 Michigan State University, USA; 2 University of Missouri, Columbia, USA; 3 Furtwangen University, Germany

**Keywords:** Person-oriented research, exploratory factor analysis, confirmatory factor analysis, structural equation model, latent class analysis

## Abstract

In this article, we demonstrate that latent variable analysis can be of great use in person-oriented research. Starting with exploratory factor analysis of metric variables, we present an example of the problems that come with generalization of aggregate-level results to subpopulations. Oftentimes, results that are valid for populations do not represent subpopulations at all. This applies to confirmatory factor analysis as well. When variables are categorical, latent class analysis can be used to create latent variables that explain the covariation of observed variables. In an example, we demonstrate that latent class analysis can be applied to data from individuals, when the number of observation points is sufficiently large. In each case of latent variables analysis, the latent variables can be considered moderators of the structure of covariation among observed variables.

## Introduction

Person-oriented research proceeds from the assumption that individuals differ in elements of structure, process, and development that are of interest in the empirical sciences (Bergman & Magnusson, 1997; von Eye & Bergman, 2003; Bogat et al., 2016). Because of these differences, statements that are based on aggregated data often cannot be defended when they are used to describe individuals and their development (for examples, see Molenaar, 2004; Molenaar, & Campbell, 2009; von Eye & Bergman, 2003).

Differences and change can be found in any parameter (cf. von Eye, 2010). For example, individuals differ quantitatively in height, weight, intelligence, music preference, food preference, or cognitive style, and individuals can differ in how these measures change over time. Individuals also differ qualitatively. For example, individuals can differ in the factor structure of intelligence, and these differences vary over the course of development (Breit et al., 2021). For example, it has been found that the number of factors needed to satisfactorily describe intelligence increases with the ability and age of children. This is known as Wewetzer’s intelligence divergence hypothesis (Wewetzer, 1958). Lienert and Crott extended this hypothesis and proposed that the number of factors decreases in late adulthood (Lienert & Crott, 1964). This is known as Lienert’s intelligence convergence hypothesis.

Still, questionnaires in Education, Sociology, or Psychology as well as psychological tests are, to this day, constructed and validated for populations rather than individuals, and often under the assumption that the structure of responses is invariant over time. Age is rarely taken into account (exceptions include tests of intelligence; see McArdle et al., 2002). Representativity is established with reference to populations of data carriers, and test-retest reliability often is determined under the assumption that development and change do not take place. By implication, questionnaires and tests may not be valid for subpopulations and they may lose their validity when respondents proceed in their development.

An example of heterogeneity in response patterns can be seen in a survey of self-perceived health (Karim et al., 2023). In this study, the authors used latent class analysis to identify groups that differ in their evaluations of such health attributes as mobility, self-care, ability to perform usual activities, pain/discomfort, and anxiety/depression. Results suggested lack of homogeneity of responses in the sense that three latent classes of responders emerged. Another example of lack of population-wide validity of a questionnaire was discussed by Wiedermann and von Eye (2016). The authors asked whether a population of students is homogeneous in their answers to an alcohol consumption questionnaire. Results from latent class analysis suggested that several subpopulations exist. In addition, results from Configural Frequency Analysis (von Eye & Wiedermann, 2022) suggest that the postulate of item homogeneity is violated in different ways in these subpopulations. In the study by Karim et al. (2023) differential item homogeneity was not investigated. Still, in both of these and in many other cases, placing individuals on dimensions of tests and questionnaires that were created to represent entire populations may be hazardous.

Statistical methods have been discussed to prevent differential item functioning from invalidating scales and tests. These methods mostly include latent growth curve modeling (McArdle et al., 2002) and alternative approaches to test theory. Molenaar (2004) showed that classical test theory cannot be used to construct tests that allow one to measure change over time. von Eye et al. (2015) discuss problems with differential item functioning in Rasch modeling. Nesselroade and Molenaar (2022) propagate the idiographic filter and propose replacing standardized measurement with this filter. In a similar context but unrelated to test theory, logic-based approaches have been proposed to deal with unexplained heterogeneity (Adamčík, 2017).

In all of these discussions, it is considered important to specify dimensions of such constructs as intelligence or response patterns such that they are valid for groups of individuals, particular periods of development, or particular social or geographical contexts. That is, researchers consider measurements that can reflect dimensions that vary with subpopulation, developmental stage, or context. If such measurements can be established, the chief postulate of person-oriented research is fulfilled, according to which sub-populations, even individuals and their development can be described only with custom-tailored measures. Dimensions are unique and cannot be described with measures that were derived from aggregated data.

Depending on the degree to which research has made progress, these subpopulations, groups of individuals, or individuals are either known or need to be identified. Research with known subpopulations has a confirmatory component. Research aimed at identifying such subpopulations has an exploratory component.

In this article, we propose using latent variable models for both confirmatory and exploratory person-oriented research. To this aim, we discuss exploratory factor analysis, structural equations modeling and latent class analysis. The focus is on categorical moderators (latent variables). Metric moderators are conceivable. However, to identify distinct groups, the subpopulations, categorical moderators seem more useful.

The remainder of this article is structured as follows. We first describe exploratory factor analysis and confirmatory structural equation models with categorical moderator variables. Then, we describe latent class analysis. This is done in an exploratory context. The application of each of the methods is illustrated with real-world data.

## Latent variable models

### Factor models

In the following section, we first consider exploratory (EFA) and then confirmatory factor analysis (CFA). For the former, consider the factor model


X=Fw+∈,

where the factor matrix *F* is *q*-dimensional, the covariance (or correlation) matrix *X* is *p*-dimensional, the matrix of weights or factor loadings w is *q* x *p*-dimensional, and *ϵ* is *p*dimensional (see Arminger et al., 1995; Bartholomew & Knott, 1999). The vector *ϵ* contains the residuals. These are assumed to be uncorrelated with the latent variables *F* and with each other. This factor model represents the linear relation between the observed values in *X* and the weighted latent variables in *F*. The residuals represent measurement error in *X* and model imperfections.

When the assumptions concerning *ϵ* are justifiable and the factor model explains the covariations among the observed variables, the covariance matrix of the *ϵ* is a diagonal matrix, *ψ*, and the covariance matrix of *X* is


$$Var(X)=w^{T}Var(F)w+\psi .$$

When the latent variables are uncorrelated and have unit variance, the covariance matrix of X becomes


$$Var(X)=w^{T}w+\psi .$$

All this applies when the data analyst estimates model parameters under the assumption that there exists just one population and that the population parameter estimates are valid for every member of this population. Taking a person-oriented perspective, this cannot be more than a first step, because this perspective allows the presupposition that distinct subpopulations exist. When these subpopulations are known, one can ask whether they differ in important parameters. In the present context, we ask whether they differ in their factor structures or loading patterns.

A first step in the direction of studying factor structures in person-oriented research can involve performing standard exploratory factor analysis (EFA), separately for each of the hypothesized groups, and, then comparing the solutions. For *J* subpopulations, *J* solutions will result. To obtain a first idea of whether these *j* solutions differ, visual inspection may suffice. The solutions are


$$X_{j}=F_{j}w_{j}+\in_{j},$$

where *j* = 1, …, *J*.

### Data example

For the following example, we use data from a study on customer satisfaction (Lobato-Calleros et al., 2007). In Mexico, the government provided milk for low-income segments of the population. 266 individuals answered questions on their expectations concerning the milk supply program (abbreviated by Expec), ease of use (Facilid), efficiency of distribution (Eficien), usefulness of program (Utilid), perceived quality of the distributed milk (Calidp), satisfaction with the program (Satis), and their trust in this program (Confi). All answers were recorded on a scale from 1 through 10, with 1 indicating low values. One of the questions concerned the distance of the milk distribution post from home. The answers were coded as (subjectively) close = 1 and far = 2. We now ask whether the factor structure of the answers varies with distance from distribution post. Each of the distance groups contains 133 respondents.

To answer this question, we perform one EFA each for the two groups. Specifically, based on correlations, we estimated two-factor solutions of maximum likelihood factor analysis. The solutions were oblimin-rotated. Table 1 displays the rotated pattern matrices for the near and the far distance groups.

**Table 1:** Rotated pattern matrices (EXPECT = expectations, FACILID = ease of use, EFICIEN = efficiency, UTILID = usefulness, CALIDP = perceived quality, SATIS = satisfaction).

	Near distance group		Far distance group	
	Factor 1	Factor 2	Factor 1	Factor 2
EXPECT	0.271	-0.043	-0.047	0.500
FACILID	-0.059	0.587	0.002	0.214
EFICIEN	0.180	0.768	0.793	0.435
UTILID	0.404	0.357	0.457	-0.066
CALIDP	0.818	0.099	-0.106	0.738
SATIS	0.577	0.343	0.209	0.669
CONFI	0.386	0.347	0.046	0.658

In the near distance group, Usefulness, Perceived Quality, and Satisfaction ratings have the strongest loadings on the first factor. Ease of Use and Efficiency show the strongest loadings on the second factor. In the far distance group, Efficiency and Usefulness load strongest on the first factor. Perceived Quality, Satisfaction, and Trust load strongly on the second factor. In other words, in the two distance groups, Efficiency and Perceived Quality load on different factors.

### Confirmatory multi-group factor models

Clearly, the two analyses on the distance subgroups of the population of Mexican milk recipients point at group differences. If these differences can be statistically confirmed, an analysis of the entire sample (not reported here) might be pointless. To statistically determine whether differences exist, we consider multi-group structural factor models.

Let, in LISREL notation (Jöreskog & Sörbom, 2001), the assumed model be


x=∧ξ+δ,

where *ξ* are the factor loadings and *δ* are the residuals, *E*(*ξ*) = 0, *E*(*δ*) = 0, and the residuals are uncorrelated with the factors. Now, let Φ=*E*(*ξξ^T^*) be the matrix of factor intercorrelations and Θ=(𝛿𝛿^𝑇^) the covariance matrix of the residuals. Then, the covariance matrix Σ of the observed variables is


$$\Sigma =\wedge \Phi \wedge^{T}+\Theta .$$

Given sufficient degrees of freedom, this relationship can be tested statistically. The tests are the well known goodness-of-fit tests. In the present context of person-oriented research, we are interested in the comparison of multiple groups, that is, in multi-group analysis. In the null hypotheses of multigroup analysis, it is posited that the parameter matrices of the groups that are studied are equal.

Let *g* be the number of groups. Then, sample null hypotheses include


$$\begin{array}{c} \Phi^{1}=\Phi^{2}=\ldots =\Phi^{g},\\ \Lambda_{x}^{1}=\Lambda_{x}^{2}=\ldots =\Lambda_{x}^{g},\\ \text{or}\\ \Lambda_{y}^{1}=\Lambda_{y}^{2}=\ldots =\Lambda_{y}^{g}, \end{array}$$

where the Φ are the matrices of factor correlations, the Λ*_x_* are the loading matrices of the x variables (i.e., the exogenous variables of the model), and the Λ*_y_* are the loading matrices of the *y* variables (the endogenous variables of the model). Any other parameter matrix can be compared, and that either alone or in combination with comparisons of other parameter matrices. This includes the model in which all parameters are compared.

### Data example (cont.)

We now resume the data example with the milk distribution in Mexico. To compare the factor solutions of the two distance groups (living near vs. far from milk distribution post), we first test a model on the *x*-side, in which each observed variable is the sole indicator of a latent variable, the factor loadings are free, and the residual matrix is a zero matrix. In addition, this model was specified with the constraint that Ψ^1^ = Ψ^2^, that is, that the covariances of the seven factors are identical in the two groups. The model was estimated with generalized least squares, and the admissibility check was turned on (this last specification applies to the following models as well).

This model failed to fit. Specifically, the overall goodness-of-fit Chi square was 82.23 (*df* = 28; *p* < 0.001), RMASEA = 0.12, and NFI = 0.88. The two distance groups contributed to about equal parts to the overall Chi square. This model could possibly have been improved. However, it does not reflect the factor structure that was suggested by the exploratory factor analyses.

Therefore, we now estimate a two-factor model that does reflect this structure. In this model, we only pose the constraint that Ψ^1^ = Ψ^2^, that is, that the covariance of the two factors is identical in the two groups. In addition, we neither kept the factor loading pattern unchanged over the comparison groups, nor did we constrain the indicators to show the same loadings in the two groups. The loading patterns were specified as shown in the following two loading pattern matrices.


$$\begin{array}{cc} \Lambda_{x}=\left[\begin{array}{cc} \dot{0} & \dot{1}\\ 0 & 1\\ 1 & 1\\ 1 & 0\\ 1 & 0\\ 1 & 0 \end{array}\right] & \Lambda_{x}=\left[\begin{array}{cc} \dot{0} & \dot{1}\\ 0 & 1\\ 1 & 1\\ 1 & 1\\ 1 & 0\\ 1 & 0 \end{array}\right] \end{array}$$

These matrices show that, in both groups the variables Usefulness, Perceived Quality, Satisfaction, and Trust load on the first factor, and Expectations, Ease of Use load on the second factor. In both groups, Usefulness is also allowed to load on the second factor. This is indicated by the ‘1’ on the fourth position in the second column. Perceived Quality is also allowed to load on the second factor, but only in the far distance group. The dots in positions 1 1 and 1 2 indicate that these loadings were fixed to 1. This loading pattern reflects the results from exploratory CFA of the first, the near distance group. Finally, the model was specified with the constraint that Ψ^1^ = Ψ^2^, that is, that the covariance of the two factors is identical in the two groups. This model was also estimated with generalized least squares.

After freeing the residual covariances between Usefulness and Effectivity and Perceived Quality and Ease of Use in the far distance group, this model fit the data well. Specifically, a global goodness-of-fit Chi-square of 18.02 (*df* = 22; *p* = 0.70) was estimated, and we obtained RMSEA = 0.016 (0.000 ≤ RMSEA ≤ 0.080). Both of these values suggest tentatively retaining the model.

Equally important is that this two-group model describes both distance groups well. The goodness-of-fit index GFI was 0.98 in both groups. The standardized RMR was 0.036 the first group and 0.06 in the second.

There are, however, two major issues. First, the residual plots differ. In the first group, the plot is near perfect. All residuals are extremely near the optimal line, and there is no extreme value. In the far distance group, however, each and every residual lies below the optimal line. That is, all values for this group were overestimated. From this, we conclude that the model performs better for the near distance group than for the far distance group. Considering that the confirmatory factor model was tailored to reflect the exploratory factor solution of the near distance group, this result does not come as a surprise.

Second, and even more importantly, none of the loadings is significant, and that in both groups. The model thus suggests that the two factors do not sufficiently capture the covariance of the seven indicator variables. The factor structure that reflects the results of EFA for the near distance group is, therefore, considered not statistically confirmed. The groups differ and the exploratory two-factor structure is, from a confirmatory perspective, invalid.

This result is important for person-oriented research. It suggests that

1. the seven indicators used in the Mexican consumer satisfaction study of the state-provided milk distribution program cannot be depicted by a factor model for the entire population; and
2. the two-factor solution that was derived from an EFA describes the covariations among the seven indicators of this study very well, but
3. it also suggests that the seven indicators are unrelated to the two factors;
4. although the model fits well, the two groups differ in that
  - to fit well, two residual covariances the far distance group had to be estimated, and
  - the residual distribution of the second group points at overestimation of covariances throughout.

In sum, the present results suggest that the result from EFA cannot be confirmed and that, in this example, group differences exist to the extent that a joint factor structure cannot be established. We conclude that the near and the far distance groups respond to the consumer satisfaction question in such a manner that they differ quantitatively and qualitatively. This exemplifies, again, that generalizations from solutions that are based on aggregated data can be hazardous.

### Latent variable models at the level of the individual

In the following paragraphs, we continue our journey from aggregate-level latent variable modeling to the modeling of subgroups of respondents and now to modeling data from individuals. As was discussed in detail in the literature (e.g., Molenaar, 2004; von Eye & Bergman, 2003), and as was illustrated here again, aggregating data before data analysis can lead to results that cannot easily be generalized to the individual. Estimating parameters at the individual level first and then aggregating parameters is often recommended (Molenaar & Campbell, 2007). For this to be possible, a sufficient volume of data is needed that allows one to perform parameter estimation at the level of the individual. This applies in particular to the number of observations that are subjected to data analysis.

Consider, for example, factor models of the kind discussed in the previous sections. There exists a large number of recommendations concerning the sample size for EFA. de Winter et al. (2009) suggest that, when data are well conditioned, a sample size of 50 may be sufficient. Other sources recommend sample sizes in excess of 100. For the analysis of individuals, this implies that 50, 100, or even more observations of the same variables are needed, that is, a lengthy series of repeated observations. For this volume of measures, factor models can be estimated for the individual.

These models often are longitudinal factor models as they were discussed and applied by for example Nesselroade and Molenaar (2010; cf. McArdle, 2007). The samples in such models are the longitudinal observations. Variables are defined as in standard factor analysis. Therefore, the factor model is the same as before, that is, 𝑥 = Λ𝜉 + 𝛿, where the *x* represent the observations on multiple occasions for just one person. The covariances that are analyzed in such a model indicate the degree to which variables covary over time, and the factors represent the covariation of variables.

Now, when development of individuals is examined, one can estimate exploratory or confirmatory factor models for segments of the series of observations. In this case, the number of necessary observations applies to each segment. In brief, segment-wise factor analysis requires large numbers of observations.

### Data example: The development of alcoholics

In the following example, we analyze data from a longitudinal study on the development of alcoholism (Perrine et al., 1995). A sample of male respondents who had identified themselves as alcoholics indicated in automated phone interviews, on a daily basis, the amount of alcohol they had consumed the day before. They also answered a number of questions concerning their mood and health.

Here, we analyze the responses that were given by the respondent with the ID 3000. This respondent answered the interview questions on 750 consecutive days. Here, we analyze the covariation of the questions concerning the consumption of beer, hard liquor, and wine, as well as the subjective ratings of Stress, Mood, and Health. The alcohol consumption questions were answered in units of ‘shots,’ where one shot is equivalent of 0.33 l of beer, 2 cl of hard liquor, and one glass of wine. The questions on Stress, Mood, and Health were answered on rating scales with 10 levels, were 1 indicates no stress, lousy mood, and poor health.

For the analysis of these data, we proceed as in the first example, in three steps. We start from an EFA of the entire data set of six variables and 750 observations. We then, in the second step, estimate a confirmatory factor model for the entire data set. In the third step, we analyze a hypothesis of development and estimate a confirmatory two-group factor model in which the first half of the 750 responses constitute the first group and the second half constitutes the comparison group.

### Exploratory factor analyses of the split data set

Using the same factor model as in the first two examples, we estimate separate exploratory maximum likelihood two-factor models for the two response periods. This was done to answer the question whether over the course of the first 375 days of drinking the covariation of the six variables differs from the covariation over the following 375 days. We expected the first factor to be constituted by the alcohol consumption variables (drinking of beer, hard liquor, and wine) and the second factor to be constituted by the well-being variables (Stress, Mood, and Health), both over both response periods. The maximum likelihood factor analyses were specified to result in two factors. Rotation was oblique. Table 2 displays the rotated pattern structures for the two response periods.

**Table 2:** Rotated pattern structures for first and second response periods (F1 = Factor 1, F2 = Factor 2)

	1^st^ Half		2^nd^ Half	
	F1	F2	F1	F2
BEER	-0.022	0.072	0.368	-0.074
LIQUOR	0.107	-0.065	0.128	0.036
WINE	0.030	0.260	0.019	-0.216
STRESS	-0.968	-0.138	0.222	0.595
MOOD	0.102	0.977	0.103	-0.131
HEALTH	-0.184	0.361	1.021	0.017

In the first response period, Stress is the only variable with a strong loading on the first factor, and Mood is the only variable with a strong loading on the second factor. None of the alcohol consumption variables has any strong loading on either factor. In the second response period, Health is the only variable with a strong loading on the first factor, and Stress is the only variable with a strong loading on the second factor. Again, none of the alcohol consumption variables has any strong loading on either factor.

Based on these results, we conclude first that our expectation that there exist alcohol consumption and well being-related factors was thwarted. Still, there are hints at possible changes in the covariation structure. The importance of Mood in the first response period fails to show up in the second, but the importance of stress shows up in both response periods. Alcohol consumption seems to be of no importance in either response period. As was obvious in the first two examples, however, the results from EFA are not necessarily indicative of results from CFA. Therefore, we now proceed and perform confirmatory factor analyses.

### Confirmatory multi-factor models

The first model that we estimate here serves to determine whether the covariance matrices remain unchanged over the two observation periods. The model is specified as follows:

1. there are six factors, constituted by one variable each; these are the three alcohol consumption and the three well-being variables; that is, the matrix of loadings is an identity matrix;
2. the response periods serve as a two-category moderator;
3. the matrix of residuals of the observed variables is a zero matrix, and
4. the matrix of factor intercorrelations is unchanged over the two categories of the moderator (matrices were fixed to be equal)
5. estimation is done with maximum likelihood and the admissibility check is turned on.

The overall goodness-of-fit Chi square for this model suggests rejecting it (Chi square = 110.99; *df* = 21; *p* < 0.001). Rejection is also suggested by the RMSEA of 0.10. Both groups contributed about equally to the overall Chi square (43% and 57%, respectively). The normed fit index, NFI, was 0.65, that is, rather low. In contrast, the overall goodness-of-fit indices are high for both response periods (GFI1 = 0.97; GFI2 = =.93).

From these results, we conclude that the covariance matrices of the two response periods are unequal, and we move to estimate a confirmatory two factor model that reflects the results of the exploratory analyses and the hypotheses about the constitution of the two factors. These hypotheses resulted in the following loading pattern matrix:


$$\Lambda_{1}=\Lambda_{2}=\left[\begin{array}{cc} 1 & 0\\ 1 & 0\\ 1 & 0\\ 0 & 1\\ 0 & 1\\ 0 & 1 \end{array}\right]$$

where the subscripts of the Λs indicate the response periods, the first three indicators are the alcohol consumption variables, and the second three are the well-being variables. In all, the two-factor moderated confirmatory factor model was specified as follows:

1. there are two factors that are patterned as given above;
2. this specification remains unchanged over the two response periods;
3. the factor loadings are freely estimated for each response periods, but the patterns are the same; that is, they are fixed to be unchanged over the two response periods;
4. the factor correlation is free but fixed to be unchanged over the two observation periods;
5. estimation was done with maximum likelihood and the admissibility check was turned on.

**Figure 1:**
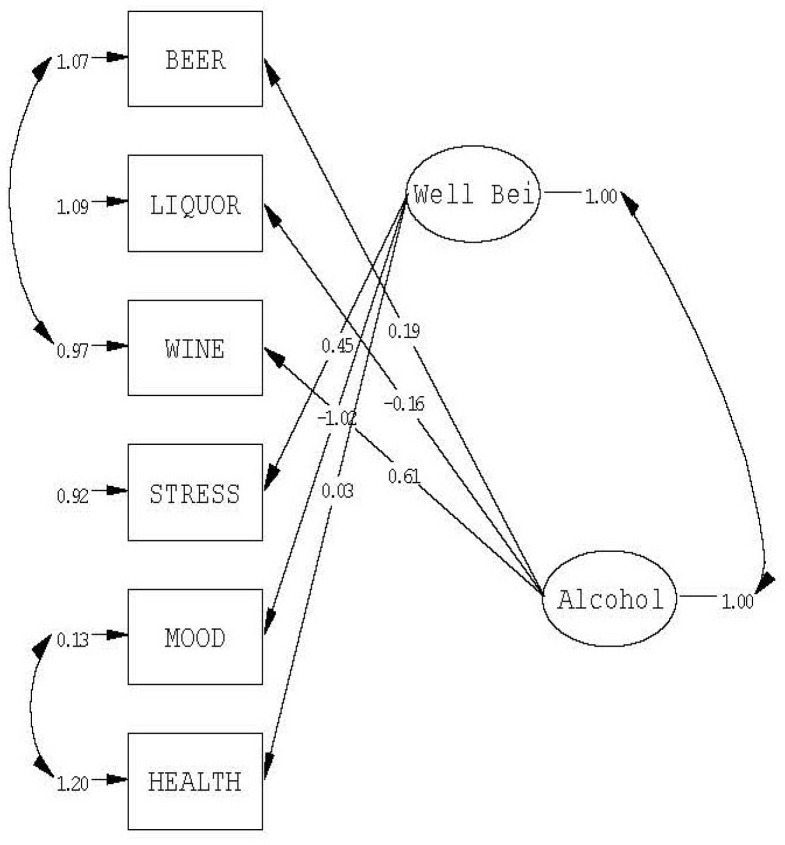
Confirmatory factor model for first response period (standardized solution)

This model did converge but it fit poorly (details not reported here). Therefore, based on the modification indices and on theory, residual covariances and cross-loadings were set free. This was done without altering the specifications that the factor correlations and the original loading patterns are unchanged over time. The resulting model fits well. We obtained an overall goodness-of-fit Chi square of 58.49 (*df* = 19; *p* < 0.001), but an RMSEA of 0.069 (95% CI 0.048 – 0.092; this confidence interval includes the value of 0.05, that is, the value for ‘close fit’). The GFI for both response periods is 0.98. The first response period contributes 37% to the overall chi square, and the second response period 63%. Figure 1 displays the final model for the first response period.

The graph for the second response period (not given here) is very much the same as for the first. The loadings are, as specified, the same. In both, the loading of Health on the Well Being Factor is non-significant. The models differ only in the residual covariances that were set free to improve model fit. In the first response period, the residual covariance Wine-Beer was estimated. This correlation is negative, suggesting that, when Case 3000 drank beer, he did not drink wine. In the second response period, the covariance Health-Mood was set free. This correlation is positive, suggesting that good mood goes hand in hand with self-rated good health.

Overall, this result suggests that, first, during the first 375 days of responding to the phone interview, the covariation of alcohol consumption with self-rated well-being is largely the same as the covariation of alcohol consumption with self-rated well-being during the second 375 days of responding. Minor differences can be seen in within-factor covariations. This result suggests that, again, the outcomes of global EFA can provide poor guidance for the specification of person-oriented moderated EFA. Finally, all examples given thus far illustrate that moderated factor analysis, be it exploratory or confirmatory, can yield results that are well in line with person-oriented research, and that generalizations of results from aggregated data to subpopulations or individuals can be invalid.

Thus far in the discussion of moderated latent variables models, we proceeded from the assumptions that moderator variables exist (or were specified based on results from EFA). The questions that were asked concerned differences in factor structures between moderator categories. In addition, questions that concern developmental constancy and change were asked and answered with respect to existing segments of time. In the following paragraphs, we discuss moderated latent variables models for the case in which moderator variables do not exist a priori. We discuss this for the case in which all variables are categorical. The method of analysis that is employed is Latent Class Analysis (LCA; when variables are metric, this method is termed latent mixture modeling; cf. also latent profile analysis).

### Latent Class Analysis in person-oriented research

Moderated latent variables analysis is performed to explore or test the assumption that parameters differ across unobserved segments or categories of moderator variables. When variables are categorical, this approach to data analysis is termed *Latent Class Analysis* (LCA; Lazarsfeld, 1950; for overviews, see Clogg, 1995; Vermunt, 2010; for recent applications, see Karim et al., 2023, Wiedermann & von Eye, 2016; for the following, see also Clogg & Goodman, 1985).

Based on Clogg (1995, pp. 317 – 318; see also Vermunt, 2010), the latent class model for categorical variables can be described as follows. Let *y* indicate a cell of the cross-classification of *J* observed variables, and let *p_Y_* (y) be the joint density of these variables. In Configural Frequency Analysis (von Eye & Wiederman, 2022), *y* would be called a *Configuration*, in other contexts of categorical data analysis, *y* often is called a *pattern* (cf. Agresti, 2012). Let *X* be the unobserved categorical moderator, that is, a latent variable. Then, the density for Category *t* of *X* is 𝑃(𝑌 = 𝑦 ∣ 𝑋 = 𝑡). According to the axiom of local independence, one obtains


$$\pi_{Y|X(t)}(y)=\Pi_{j=1}^{J}\pi_{Y}{}_{{}_{j}|X(t)}(y_{j}).$$

When local independence prevails, the latent class model explains the covariation among the observed variables (for an application of Configural Frequency Analysis to test whether local independence applies, see Wiedermann, & von Eye, 2016). This is in analogy to factor analysis. The joint distribution of Y and X,𝜋_𝑌,𝑋_(𝑦, 𝑡), is


$$\pi_{Y,X}(y,t)=\pi_{x}(t)\pi_{Y|X(t)}(y),$$

and the latent class model is


$$\pi_{Y}(y)=\Sigma_{t=1}^{T}\pi_{Y,X}(y,t).$$

Alternatively, the model can be expressed in terms of loglinear models (see Clogg, 1995; Clogg & Goodman, 1985).

In exploratory model search, results often include multiple fitting models. Models fit when goodness-of-fit tests suggest no significant model-data discrepancies. In latent class analysis, the likelihood ratio Chi square test is most often used. To compare models, information criteria can be used. In either case, comparatively small values point at preferable models (Agresti, 2012; Clogg, 1995).

### Data example: Structure of responses given by an alcoholic (cont.)

We now revisit the data example of the case labeled 3000 who had provided answers to phone interview questions over an uninterrupted period of 750 days. The rating scales used in this interview had 10 scale points, and the alcohol consumption questions were simply the number of beers, glasses of wine or shots of hard liquor reported for a day. The rating scale points could in principle be crossed without rescaling. However, to be able to use multiple rating scales, that is, to avoid large, sparse cross-classifications, and to be able to relate them to alcohol consumption, we transformed the scales to be used in this example as follows:

1. The scales for Stress and Mood were rescaled into three ordinal categories as
  - 1 = original 1, 2, and 3
  - 2 = original 4 and 5, and
  - 3 = original 6 and above;
2. The scale for Health was rescaled into two ordinal categories as
  - 1 = original 1 and 2
  - 3 = above original 2;
3. the consumption figures for beer, hard liquor, and wine were rescaled into two categories as
  - 1 = original zero (no consumption reported), and
  - 2 = one or more units of consumption reported.

Crossed, these five variables span a 3 × 3 × 2 × 2 × 2 table. The question we are trying to answer with the data in this table concerns the structure of the 750 responses. Specifically, we ask whether one latent variable can be found that has only a small number of categories within which responses are independent. In other words, we ask whether the covariation of the answers given by this self-declared alcoholic belongs to one of a small number of answer classes. These classes and their number are unknown before analysis. The cross-classification of the five variables to be analyzed is given in Table 3.

**Table 3:** Stress × Mood × Health × Beer × Liquor × Wine cross-classification. Values under the first five variable names indicate variable levels; the last two columns indicate observed frequencies of the two variable levels for Wine.

Stress	Mood	Health	Beer	Liquor	Wine	
					1	2
1	1	1	1	1	20	0
				2	3	0
			2	1	3	0
				2	1	0
		2	1	1	2	1
				2	2	0
			2	1	0	0
				2	0	0
	2	1	1	1	5	0
				2	5	0
			2	1	13	0
				2	4	0
		2	1	1	15	2
				2	13	2
			2	1	30	3
				2	20	1
	3	1	1	1	2	1
				2	0	1
			2	1	1	1
				2	1	0
		2	1	1	1	4
				2	5	3
			2	1	8	7
				2	4	1
2	1	1	1	1	10	1
				2	1	0
			2	1	2	1
				2	2	0
		2	1	1	3	1
				2	3	0
			2	1	4	0
				2	4	0
	2	1	1	1	12	5
				2	9	2
			2	1	15	2
				2	5	1
		2	1	1	49	7
				2	14	3
			2	1	59	5
				2	20	5
	3	1	1	1	0	0
				2	0	0
			2	1	1	0
				2	0	0
		2	1	1	1	2
				2	0	1
			2	1	1	0
				2	0	1
3	1	1	1	1	9	0
				2	3	0
			2	1	5	0
				2	1	0
		2	1	1	11	4
				2	11	0
			2	1	12	3
				2	10	2
	2	1	1	1	14	0
				2	7	1
			2	1	14	0
				2	1	0
		2	1	1	47	7
				2	27	1
			2	1	78	5
				2	32	1
	3	1	1	1	1	0
				2	0	0
			2	1	0	0
				2	0	0
		2	1	1	1	0
				2	1	0
			2	1	2	0
				2	2	0

*Note*. The 72 rows represent the 72 possible value patterns of the first five variables, Stress × Mood × Health × Beer × Liquor (3 × 3 × 2 × 2 × 2 = 72). For the sixth variable, Wine, each of its two value levels (1 and 2) has a separate column, indicating the observed frequencies of each specific value pattern.

In a preliminary step, we estimated a log-linear main effect model for the cross-classification in Table 3. The goodness-of-fit LR Chi square for this model is 343.500 (*df* = 135; *p* < 0.001). The model can, therefore, be rejected, and we conclude that the five variables covary.

To investigate the dependence structure, we estimate latent class models. Goal of the analysis is the explanation of the covariation of the variables in Table 3. This covariation is considered explained when subgroups can be identified within which there is no covariation left (local independence). We estimate a model with the following characteristics:

1. the subgroups that are searched for are represented by one latent variable; the number of categories of this variable is subject to exploration;
2. the latent distribution can vary over the categories of the latent variable;
3. the conditional probabilities can vary over the categories of the latent variable.

Because of the second and the third characteristic, this model is *completely heterogeneous*. In a slightly simplified notation, the model is


$$\pi_{SMHBLWG}=\pi_{G}\pi_{S|G}\pi_{M|SG}\pi_{H|SG}\pi_{B|SG}\pi_{L|SG}\pi_{W|SG},$$

where S, M, H, B, L, and W are the first characters of Stress, Mood, Health, Beer, Liquor, and Wine, and G indicates the latent variable.

To identify the best latent class model for the data in Table 3, we estimated models in which the sole latent variable has 2, 3, and 4 categories. The overall goodness-of-fit scores for these models are given in Table 4. In addition to these values, we inspect, for model selection, the residual distribution. We select models with small numbers of extreme residual values.

**Table 4:** Goodness-of-fit scores for three latent class models of the Well Being – Alcohol Consumption data of the respondent labeled 3000.

Goodness-of-fit score	Number of categories of latent variable		
	2 categories	3 categories	4 categories
Pearson Chi square	144.887 (*df* = 103; *p* = 0.003)	67.393 (*df* = 81; *p* = 0.86)	28.263 (*df* = 60; *p* = 0.99)
LR Chi square	126.034 (*df* = 103; *p* = 0.054)	68.845 (*df* = 81; *p* = 0.83)	40. 573 (*df* = 60; *p* = 0.97)
BIC	-549.21	-467.38	-356.63
AIC	-77.97	-93.16	-79.43

Based on the results shown in Table 4, we retain the model in which the latent variable has three categories (latent classes). Using only the LR Chi square, the model with two latent classes could have been retained as well. However, the residual distribution of this model includes eight cells with residuals greater than 2.0 and one cell with a residual greater than 3.0. In contrast, the solution with three latent classes contains only two cells with a residual value greater than 2.0. Considering both, the Chi square measures and the information criteria, the model with four latent classes fits better than the one with three categories. However, it is less parsimonious, there is still one cell with a residual greater than 2.0, and there were estimation problems with a number of parameters. Therefore, again, we retain the model with three classes.

To interpret the three latent classes, we use the probabilities with which profiles of the six observed variables in the model can be found in the three classes. This is in analogy to the interpretation of factor loadings to label factors in EFA. Table 5 presents these probabilities. We consider variable categories greater than 0.55 markers for the classes of the latent variable.

**Table 5:** Probabilities of the three-category LCA solution for the observed six well-being and alcohol consumption variables (markers printed in bold)

Category of latent variable			
	G=1	G=2	G=3
p(C)	0.2734	0.5870	0.1396
S=1	0.1317	0.2854	0.3089
S=2	0.2937	0.3857	0.2098
S=3	**0.5747**	0.3288	0.4814
M=1	0.0993	0.1530	0.4516
M=2	**0.7837**	**0.7917**	0.4946
M=3	0.1170	0.0554	0.0538
H=1	0.2458	0.1292	**0.7518**
H=2	**0.7542**	**0.8708**	0.2482
B=1	0.4539	0.4356	**0.6799**
B=2	**0.5461**	**0.5644**	0.3201
L=1	**0.7043**	**0.6586**	**0.7512**
L=2	0.2957	0.3414	0.2488
W=1	**0.7950**	**0.9455**	**0.7902**
W=2	0.2050	0.0545	0.2098

*Note*. S = Stress; M = Mood; H = Health; B = Beer; L = Liquor; W = Wine

The first row under the headers in Table 5 suggests that the first latent class has a probability of *p* = 0.27. The second latent class is the greatest, with *p* = 0.59. The third latent class is the smallest, with *p* = 0.14. Responses that represent the first latent class have the profile *high stress, average mood, good health, high beer consumption, low hard liquor consumption*, and *low wine consumption*. The second latent class has the markers *average mood, good health, high beer consumption, low hard liquor consumption*, and *low wine consumption*. Evidently, the second latent class differs from the first only in the absence of stress categories as markers. The third latent class differs greatly from the first two. Its markers are *poor health, low beer consumption, low consumption of hard liquor*, and *low wine consumption*.

In more colloquial terms, responses that belong to the first latent class represent situations in which Case 3000 experiences high stress, is not in the worst of moods or poor health, and drinks large amounts of beer, but no other alcoholic beverages. Responses that belong to the second latent class represent situations in which Case 3000 drinks copious amounts of beer even without increased stress. This is, based on the observed 750 days, the most likely pattern. The least likely responses – they belong to the third latent class – are given when Case 3000 feels like he is in poor health and abstains from drinking large amounts of alcohol of any sort.

Considering the impressive goodness-of-fit scores of the present model, we also estimated a model in which specific constraints were placed. More specifically, we estimated the model


$$\pi_{SMHBLWG}=\pi_{G}\pi_{S|G}\pi_{M|G}\pi_{H|G}\pi_{B|G}\pi_{L|G}\pi_{W|G}.$$

This model proposes that the measurement model is the same across the categories of the latent variable. Under this constraint, model fit is mediocre, at best. We obtain X-squared = 139.988 (*df* = 117; *p* = 0.073), LR X-squared = 149.235 (*df* = 117; *p* = 0.024), BIC = -625.314, and AIC = -84.765. Even if this model is considered retainable, the solution suffers from five residuals that are greater than 2.0 and two that are greater than 3.0. Therefore, we stay with the above solution.

Alternative models could have been considered. These models include, for example, those that constrain heterogeneity to particular categories of the latent variable, or models with multiple latent variables. These models can be estimated in future work, in particular when theories exist that would allow one to specify particular and partial constraints.

## Discussion

In this article, we point researchers to the option of estimating latent variable models in person-oriented data analysis. We discuss and illustrate exploratory and confirmatory factor analysis (EFA and CFA) with metric variables. In examples, we demonstrate that both EFA and CFA can be estimated at the aggregate level, but also at levels that represent subpopulations and that allow one to compare subpopulations. The examples also show that solutions at finer-grained levels can differ dramatically from solutions at the aggregate level. Group comparisons are possible as well, with respect to any model parameter.

When variables are categorical, latent class analysis (LCA) can be performed, with the same aims. Using LCA as a sample method, we illustrate that modeling can be performed even at the level of the individual. As in CFA, group comparisons can be modeled and longitudinal data can be analyzed.

One important element of modeling at finer-grained levels is that the size of the data body that is subjected to analysis be sufficiently large. Rules of thumb exist according to which a sample size of *n* = 100 is needed for EFA. However, for EFA as well as for structural equation models, a priori sample size calculators are available. These calculators take into account the RMSEA, the degrees of freedom, the power that the data analyst aims at, the significance threshold *α*, the number of latent variables, and the number of observed variables. In most cases, the sample better be of considerable size. Effects can usually be detected with smaller samples, as in regression analysis. To establish the factor structure, larger samples are needed.

Consider, for example, a situation similar to the one in the EFA and CFA examples above. If, in a data analysis, the anticipated effect size is 0.5, the power that the analyst aims at is 0.8, the number of latent variables is 2, the number of observed variables is 6, and the significance threshold is 0.05, then the minimum sample size to detect an effect is 23. However, to establish the factor structure, a sample size of 200 is recommended. Similarly, effect size, statistical power, and sample size studies can be used to calculate useful sample sizes for LCA (e.g., Dziak et al., 2014).

In observational studies in which samples consist of respondents (see the Mexican milk supply study, above), sample sizes of 200 or above can often be achieved. When, however, data from individuals are modeled, and sampling goes over points in time, this number of observation points may be hard to reach. Even when such simple linear models as polynomial regression are estimated for individuals the number of observation points that are needed to reach standard power levels can already be large (see von Eye & Wiedermann, 2015). Still, it is important to realize that latent variable models can be estimated at the level of the individual.

The models discussed in this article can be enriched in many respects. For example, multiple factors or latent variables can be considered. Latent variables can have three or more categories. Multiple groups can be compared in their factor structures or latent variables. Time series can be modeled. Multiple groups can be compared in their changes in factor structure or latent variables. Covariates can be considered. Latent variable path models can be estimated. In each of these and many other cases, aggregate-level models can be estimated as well as individual-level models.

Methods that also can be important in the context of identifying heterogeneity include cluster analysis (aka unsupervised classification; see, e.g., El Abbassi et al., 2021). These methods are used to identify homogeneous groups that had been unknown before data collection. In standard applications, the groups that result from cluster analysis contain, for example, respondents, stars, or responses to chemicals. Here, in the application to data from single individuals, clusters can describe behavior patterns over time segments, over occasions, or over physical environments. In other words, clustering can be applied to the same individual-level data as factor analysis, latent class analysis, and structural equation modeling. The same requirements apply.

It is important to note that, in standard application, cluster analysis and latent variable analysis utilize different data characteristics. In cluster analysis, data points in the same clusters are more similar or closer to each other than to data points in other clusters. In contrast, latent variable analysis models variable covariances. Latent variables, therefore, represent covariance structures, not distances or similarities of data points.

When the analysis is completely unsupervised, no reference is made to underlying variable distributions. It is, however, possible to take the assumption into account that variables follow specific distributions such as the normal.

By way of analogy, cluster analysis of data that describe an individual results in groups of data points that are more similar or closer to each other than to data points in other clusters in particular segments of time or location, or both. In other words, clusters of data from an individual describe similar responses in particular segments of time, occasion, or location for just one person. In contrast, latent variables from person-oriented analysis explain variable covariation that varies over time and space, for one person.

One characteristic of latent variable modeling at higher aggregate levels is that measurement error can be taken into account. This is unchanged when data from individuals are analyzed. However, it should be noted that, while structural equation modeling does take measurement error into account, this is not the case in standard applications of cluster analysis. Application of these methods proceeds under the assumption that data are error-free or that measurement errors are negligible.

In addition to estimating alternative or more complex models, statistical methods can be applied to go into more depth with the goal to examine the characteristics of factor and latent class solutions. Wiedermann and von Eye (2016) proposed using Configural Frequency Analysis (von Eye & Wiedermann, 2022) to test whether local independence holds in latent classes. Other options include comparing item distributions in latent classes or testing whether item distributions in latent classes depend on covariates, that is, observed moderators. Similarly, it could be tested whether item distributions follow a particular, a priori hypothesized form (cf., von Eye & Gardiner, 2004; von Eye & Wiedermann, 2023).

This is most important from the perspective of person-oriented research. Given the required data volume, comparisons of individuals are not restricted to visual inspection or statistical comparisons of single parameters such as means or correlations. The methods discussed here can help identify exactly where subpopulations or individuals differ, and in which parameters.

## Action editor

Lars-Gunnar Lundh served as action editor for this article.

## Open access

This article is distributed under the terms of the Creative Commons Attribution 4.0 International License (http://creativecommons.org/licenses/by/4.0/), which permits unrestricted use, distribution, and reproduction in any medium, provided you give appropriate credit to the original author(s) and the source, provide a link to the Creative Commons license, and indicate if changes were made.
